# Reversible Thermochromic Microcapsules and Their Applications in Anticounterfeiting

**DOI:** 10.3390/ma16145150

**Published:** 2023-07-21

**Authors:** Haisheng Liu, Yuhao Deng, Yang Ye, Xingqiang Liu

**Affiliations:** 1 School of Physics and Electronics, Hunan University, Changsha 410082, China; liuxq@hnu.edu.cn; 2 Materials Interfaces Center, Shenzhen Institute of Advanced Technology, Chinese Academy of Sciences, Shenzhen 518055, China

**Keywords:** microcapsule, reversible thermochromic, poly(urethane-urea), anticounterfeiting, screen-printing ink, coating

## Abstract

The common, commercial reversible thermochromic (RT) melamine–formaldehyde resin microcapsules containing formaldehyde are very harmful to human health. To address this issue, we successfully prepared a novel formaldehyde-free microcapsule via interfacial polymerization using RT compositions as the core and poly(urethane-urea) (PUU) as the shell. The core material consisted of a color former (crystal violet lactone), a developer (bisphenol AF), and a solvent (methyl stearate). To optimize the synthesis of the microcapsules, an L_9_ (3^4^) orthogonal design and single-factor experiments were employed to analyze the effects of four factors (N3300-to-L75 shell material mass ratio, core-to-shell material mass ratio, emulsifier concentration, and shear rate during emulsification) on the encapsulation efficiency. The results showed that the optimal parameter values were as follows: a shear rate of 2500 rpm, N3300-to-L75 shell material mass ratio of 1:4, core-to-shell material mass ratio of 11:5, and emulsifier concentration of 3.5%. The influence of the shear rate on the particle size and distribution, surface morphology, dispersibility, and reversible thermochromic properties of the microcapsules was investigated. Furthermore, analyses on the phase-change characteristics, thermal stability, ultraviolet aging, and solvent and acid–base resistances of the microcapsules were conducted systematically. Finally, a reversible thermochromic mark containing the RTPUU microcapsules was designed and fabricated, which could be used against falsification. Moreover, these RTPUU microcapsules can be potentially used for anticounterfeiting applications.

## 1. Introduction

**Figure 1:**
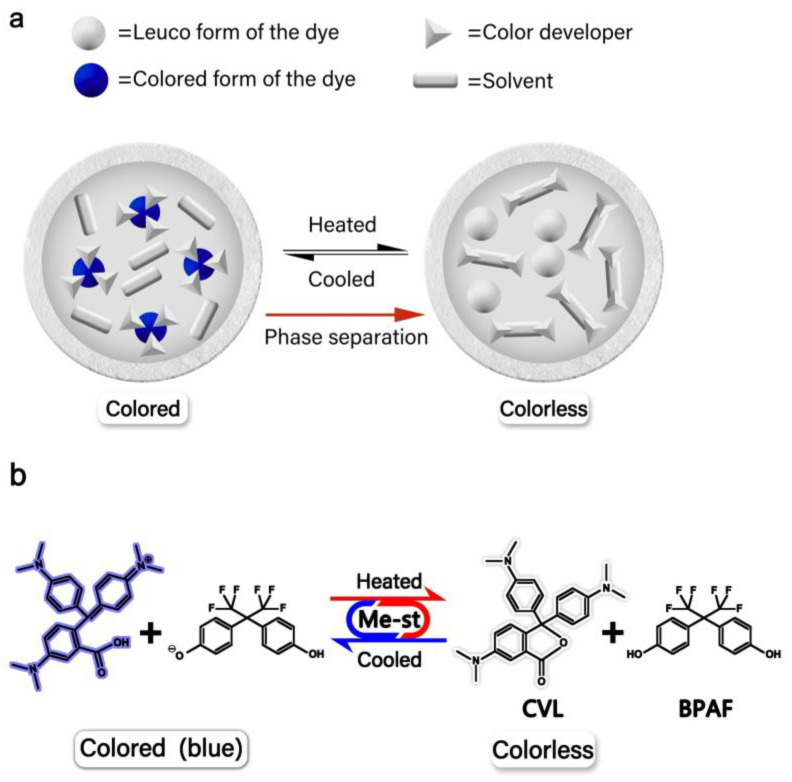
Thermochromic mechanism of RT microcapsules [10]: (**a**) Thermochromic behavior of the leuco dye/color developer/solvent composition; (**b**) Chemical formula of the CVL dye and color-forming reaction of CVL with BPAF.

Research on microcapsules with a novel reversible thermochromic (RT) function is a promising and highly popular topic, which has been generating significant commercial interest. RT microcapsules have been extensively applied in solar energy storage, wearable electronic devices, intelligent textiles, medical diagnostics, smart material coatings, and inks [1,2,3,4,5,6,7,8]. Leuco-dye-based RT compositions are widely recognized as one of the most promising organic thermochromic materials owing to their advantages of distinct and sensitive discoloration as well as low cost. Consequently, these materials have been extensively researched. Recently, leuco-dye-based RT compositions have been utilized as core materials for fabricating thermochromic microcapsules with temperature-sensitive properties [9]. Crystal violet lactone (CVL) is a leuco dye, commonly used as a color former. Bisphenol AF (BPAF) is a weak acid, used as a color developer. Methyl stearate (Me-st) is used as a solvent. As shown in Figure 1, RT compositions consist of a leuco dye (CVL), color developer (BPAF), and solvent (Me-st) [10]. When the temperature of an RT composition changes, leuco dye gains or loses protons from the color developer, and a mutual conversion between the conjugated structure and lactone ring structure of the leuco dye in the solvent manifests as a reversible color change. Specifically, at temperatures lower than the melting point of the solvent, the leuco dye gains protons from the color developer and changes its structure to that of the colored ring-opened state, where the molecules rearrange to create conjugated double bonds, enabling the dye to show color. At temperatures higher than the melting point of the solvent, the leuco dye loses protons and transforms into a colorless ring-closed state [11,12]. Consequently, the above three components of organic RT compositions, which become blue-colored under the crystallization temperature of the solvent (Me-st) and turn colorless above the melting point, were selected as the core materials of the microcapsules developed in this study.

Various methods have been developed for encapsulating RT compositions, including in situ polymerization, interfacial polymerization, suspension polymerization, emulsion polymerization, sol–gel, spray drying, and complex coacervation [13,14,15,16]. Over the past few decades, a series of RT microcapsules have been produced through in situ polymerization with melamine–formaldehyde resin as a shell material; these microcapsules have been widely commercialized because of the low cost of raw materials, simple fabrication, excellent seal tightness, and good chemical resistance. However, the release of formaldehyde due to the degradation of melamine–formaldehyde resins poses a risk to human health [17]. In contrast, no such problem is associated with microcapsules featuring a poly(urethane-urea) (PUU) shell. This study systematically discusses the encapsulation, characterization, and application of RTPUU microcapsules. The microcapsules are prepared via interfacial polymerization. The RT compositions of CVL, BPAF, and Me-st with high sensitivity and low color-changing temperature are employed as the core materials. PUU was selected as a microcapsule shell material because of its excellent chemical and mechanical properties, as well as the advantage of formaldehyde-free content compared to melamine–formaldehyde resin shells that have similar properties [18,19,20].

The objectives of this study are as follows: (1) to use the L_9_ (3^4^) orthogonal design to investigate the impact of four factors (N3300-to-L75 shell material mass ratio, core-to-shell material mass ratio, emulsifier concentration, and shear rate during emulsification) on the encapsulation efficiency and (2) to establish the optimal process for the preparation of microcapsules. Additionally, the microstructure, chemical compositions, and properties of the RTPUU microcapsules were studied. Specifically, the morphology, dispersibility, reversible thermochromic properties, phase-change characteristics, thermal stability, ultraviolet (UV) aging, and solvent and acid–base resistances of the RTPUU microcapsules were analyzed using a laser particle size analyzer, scanning electron microscope (SEM), optical microscope (OM), Fourier transform infrared (FT-IR) spectrometer, single-channel scraper fineness meter, portable colorimeter, differential scanning calorimeter (DSC), thermogravimetric analyzer (TGA), and UV-accelerated aging chamber. A reversible thermochromic anticounterfeiting mark was prepared using RTPUU microcapsules and its properties were investigated. The developed RTPUU microcapsules have a unique reversible thermal memory function and strong color contrast between the cooling and heating conditions, enabling information encryption, and they can be potentially used in anticounterfeiting, display systems, and other fields.

## 2. Materials and Methods

### 2.1. Materials

The RT compositions employed as microcapsule core materials generally contain a leuco dye CVL, color developer BPAF, and solvent Me-st. Covestro Desmodur^®^ L-75 prepolyurethane (Cheshire, UK) (a toluene diisocyanate trimethylolpropane diethylene glycol, with an equivalent weight of ca. 315, NCO content of 13.3 ± 0.4%, and nonvolatile content of 75 ± 2%) and Desmodur^®^ N3300 (Cheshire, UK) (a solvent-free aliphatic polyisocyanate HDI trimer, equivalent weight of ca. 193, and NCO content of 21.8 ± 0.3%) were employed to prepare the microcapsule shell materials. Diethylenetriamine (DETA) and Epicure 8537-WY-60 (an amine adduct of an epoxy resin manufactured by HEXION, Springfield, OR, USA) were employed as the crosslinking hardeners. CUCAT-U2 (a waterborne polyurethane catalyst from Guangzhou Yourun Corporation (Guangzhou, China)) was used to increase the reaction rate of the shell materials and crosslinking hardeners to form a poly(urethane-urea) shell. A styrene-maleic anhydride (SMA) copolymer (specifically, SMA820 from Nanjing Yinxin Chemical Corporation (Naijing, China)) was employed as the emulsifier for preparing the emulsifier solution. BASF B225 antioxidant (a synergic blend of 50% antioxidant 1010 and 50% antioxidant 168) was employed to improve the UV-aging resistance of the microcapsules. Ethyl acetate was used as the cosolvent in all formulations. All chemicals were of the technical grade, obtained from commercial sources, and used without further purification.

### 2.2. Synthesis of RTPUU Microcapsules

Preparation of the emulsifier solution (water phase): 7.0 g of SMA820 and 0.94 g of NaOH were dissolved in 200 mL of deionized water in a 1000 mL beaker and stirred using an overhead paddle stirrer at 85 °C for 1 h. After cooling, a transparent yellow liquid containing SMA was obtained and used as the emulsifier. The pH of the solution was 4.2–4.5.

Preparation of the RT compositions: RT compositions were prepared by first mixing CVL, BPAF, and Me-st in a 250 mL beaker, and then heating the mixture on a hot plate. Further, the mixture was dissolved at 130 °C to obtain a homogenous solution. Next, B225 antioxidant was added and dissolved to improve the UV-aging resistance. The RT compositions were obtained after all the substances were completely dissolved.

Preparation of the shell materials: A certain amount of Desmodur^®^ L75, Desmodur^®^ N3300, and a cosolvent (such as ethyl acetate) were added to a 200 mL beaker. The shell materials were obtained after all the substances were completely dissolved.

**Figure 2:**
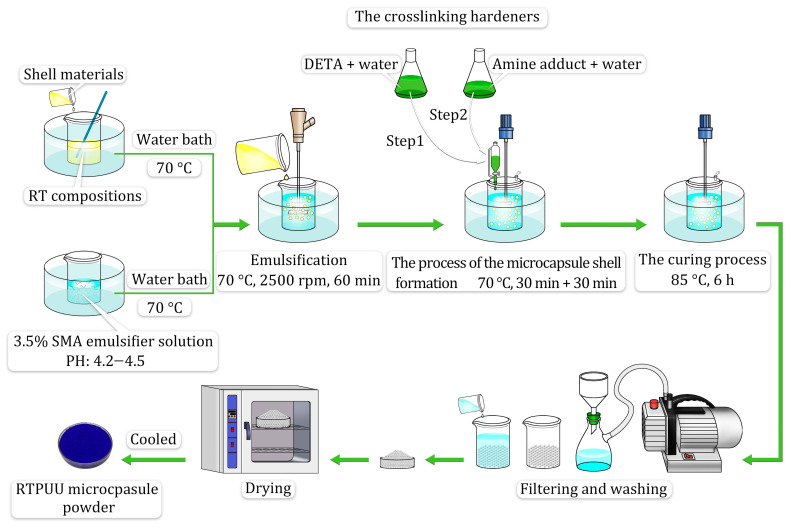
Schematic of the synthesis procedure of the RTPUU microcapsules via interfacial polymerization.

Synthesis of the RTPUU microcapsules: As shown in Figure 2, the RTPUU microcapsules were synthesized through interfacial polymerization, as described below, and the oil phase was obtained by mixing the melted RT compositions with the shell materials. The resulting oil phase was then added to the water phase and stirred using a disperser (the diameter of the dispersion disk was Φ55 mm) at a certain shear rate at 70 °C for 60 min to obtain a uniform and stable oil-in-water emulsion. The emulsion was immediately transferred to a stirring device with exhaust pipes; then, DETA and water were added. The mixture was stirred at 70 °C for 30 min. Finally, the amine adducts and water were added dropwise to the mixture, and the stirring rate was maintained at 70 °C for 30 min. After adding the CUCAT-U2 catalyst to the mixture, the temperature was raised to 85 °C, and the reaction was maintained at this temperature for 6 h. Weak acidic conditions were maintained during the reaction by adding 2 M citric acid solution to reduce the system pH to below 7. The reaction yielded microcapsule suspensions, which were recovered through filtration. Finally, the microcapsules were washed with deionized water and dried in a vacuum drying oven at 80 °C for 24 h.

### 2.3. Orthogonal Experimental Design

Based on the literature [19,20,21] and previously conducted experiments, the following four key factors that affect the encapsulation efficiency of RTPUU microcapsules were selected: (1) the N3300-to-L75 shell material mass ratio, (2) core-to-shell material mass ratio, (3) emulsifier concentration, and (4) shear rate during emulsification. Subsequently, a four-factor and three-level orthogonal experiment (Table 1 and Table 2) was designed using the Orthogonality Experiment Assistant II software (Sharetop Software Studio, V 3.1, Beijing Qingsi Technology Co., Ltd. (Beijing, China)).

In general, a full evaluation of the effect of the above factors on the encapsulation efficiency needs 81 three-level experiments. Using the L_9_ (3^4^) orthogonal design (Table 2), the number of experiments was reduced to nine. The objective was to investigate the impact of the above four factors on the encapsulation efficiency and to establish the optimal prescription component and preparation process.

### 2.4. Characterizations 

#### 2.4.1. Determination of the Actual Core Content and Encapsulation Efficiency

The amount of the encapsulated RT compositions was measured by freezing the weighted microcapsules using liquid nitrogen. Next, the microcapsules were adequately crushed using a pestle and mortar and then washed with acetone, which is a suitable solvent for RT compositions. The mortar and pestle were also washed with acetone to avoid possible measurement errors. Then, a certain amount of acetone was added to a vial containing the weighted crushed microcapsules, which were subjected to ultrasonication for 60 min. Subsequently, the mixture was carefully filtered, and the vial was rinsed with acetone to collect the residual shell material. Next, the microcapsule residuals were dried in a vacuum oven. The weight of the encapsulated RT compositions in the core was calculated as the difference between the initial weight of the microcapsules and the final weight of the dried shell residuals (crushed and washed microcapsules). The actual core content *C_a_* and encapsulation efficiency *E_a_* were calculated using Equations (1) and (2), respectively.

(1) $$C_{a}\;\left(\%\right)=\frac{M_{0}-M_{s}}{M_{0}}\times 100$$

(2) $$E_{a}\;(\%)=\frac{M_{0}-M_{s}}{M_{t}}\times 100$$

where *M*_0_ is the initial weight of the microcapsules, *M_s_* is the weight of shell residues, and *M_t_* is the weight of the fed RT compositions. 

#### 2.4.2. Particle Size Distribution (PSD) Analysis

The PSD of microcapsules was measured using a laser particle size analyzer (BT-9300ST, Chongqing Degold Machine Co., Ltd., Chongqing, China) and distilled water. The average size of microcapsules was determined based on the PSD in volume.

#### 2.4.3. SEM Analysis

The surface morphology and shell thickness of microcapsules were analyzed using an SEM (Zeiss sigma 300, Zeiss, Oberkochen, Germany). The microcapsules were set to a conductive stage and sputter-coated with a gold–palladium alloy layer before observation to minimize the charging problem.

#### 2.4.4. Optical Microscopy (OM) Analysis

The microcapsules were visually inspected using an OM in transmitted light mode along with an Olympus BX53M microscope (Olympus, Tokyo, Japan) equipped with Application Suite Interactive. Images were obtained using a digital camera attached to the microscope.

#### 2.4.5. Dispersibility Test

The dispersibility of microcapsule powder in ink was measured using a single-channel scraper fineness meter (QXP ISO 0–50 μm; Tianjin Jingke Technology Co., Ltd., Tianjin, China).

#### 2.4.6. Discoloration Temperature Estimation

The RTPUU microcapsule suspension was cooled to room temperature and coated on white matte art paper using the scratch coating method (GB 78 × 100 single-sided bird-type film applicator). The coating thickness was ~75 μm. After drying at room temperature for 2 h, the test paper coated with the microcapsule suspension was cut into small (9 cm × 6 cm) cards. The thermochromic performances of the microcapsules were investigated using a temperature control device, where an RTPUU microcapsule card of the coating was partially immersed in water. The card was sealed with a polyethylene terephthalate (PET) laminating film to prevent corrosion from water. The temperature control device was placed in a water bath and heated or cooled. As a result, the precise temperature control of the RTPUU microcapsules was achieved. The thermochromic state of the RTPUU microcapsule coating card was visually observed while changing the temperature of the water base, and temperatures *T*_1_ (complete coloration temperature), *T*_2_ (initial coloration temperature), *T*_3_ (initial decoloration temperature), and *T*_4_ (complete decoloration temperature) were recorded. The middle temperatures *T_C_* and *T_D_* during the coloring and decoloring processes, respectively, were calculated using *Tc* = (*T*_1_ + *T*_2_)/2 and *T_D_* = (*T*_3_ + *T*_4_)/2. The hysteresis width Δ*T* is an important indicator of the thermochromic performance of the RTPUU microcapsules. Δ*T* is defined as the difference between middle temperatures *T_C_* and *T_D_* (i.e., Δ*T* = *T_D_* − *T_C_*).

#### 2.4.7. Determination of Color Density and Color Residue

Here, 1.0 g RTPUU microcapsule powder was dispersed in a 9.0 g varnish used for silk screen-printing ink (SS-777, Toyo East Ocean ink) by stirring with a painting knife. After stirring and mixing evenly, the resulting mixture can be used as an ink containing 10% of the RTPUU microcapsules. Subsequently, the ink was coated on white matte art paper using a scratch coating method (GB 78 × 100 single-sided bird-type film applicator). The coating thickness was ~75 μm. After being dried in an air oven at 80 °C for 15 min, the ink coating was obtained. The color difference exhibited by the ink coating (or the suspension coating described in Section 2.4.6) was tested according to the CIELAB system using a portable colorimeter (DS-200, test mode: SCI, light source/angle: D65/10°, Hangzhou CHNSpec Technology Co., Ltd., Hangzhou, China). The color difference ∆*E* was calculated using the following equation:

(3) $$∆E=\sqrt{{\left(dL^{*}\right)}^{2}+{\left(da^{*}\right)}^{2}+{\left(db^{*}\right)}^{2}}=\sqrt{{\left(L^{*}-L_{0}\right)}^{2}+{\left(a^{*}-a_{0}\right)}^{2}+{\left(b^{*}-b_{0}\right)}^{2}}$$

where *L*, *a*, and *b* represent the color space coordinates of a specific location. *L*, *a*, and *b* represent the lightness, red–green, and yellow–blue values, respectively. L*, a*, and b* represent the lightness, red–green, and yellow–blue values of the test sample, respectively. *L*_0_, *a*_0_, and *b*_0_ represent the lightness, red–green, and yellow–blue values of a benchmark sample (i.e., a white matte art paper), respectively [22]. Color density ∆*E_C_* is defined as the color difference between the test sample at its complete coloration state and the benchmark sample. Color residue ∆*E_D_* is defined as the color difference between the test sample at its complete decoloration state and the benchmark sample.

#### 2.4.8. FT-IR Analysis

The chemical structures and compositions of the microcapsules were determined using an FT-IR spectrometer (Thermo Scientific Nicolet iS50 FT-IR, Waltham, MA, USA). The spectra were collected by averaging 32 scans at a resolution of 4 cm^−1^ in the wavenumber range of 400–4000 cm^−1^. During the test, a 0.02 g sample was dispersed in a spectroscopic grade KBr medium tablet.

#### 2.4.9. DSC Analysis

The melting and freezing behaviors of the microcapsules were investigated using a DSC (TA Instruments Discovery DSC 25, New Castle, DE, USA). Samples were heated from 0 °C to 65 °C in a nitrogen environment, then cooled back to 0 °C at a rate of 5 °C/min, and maintained at 0 °C for 5 min. The onset point of the melting or crystallization peak was considered as the phase-change temperature.

#### 2.4.10. TGA Analysis

The thermal stability of the synthesized microcapsules was investigated using a TGA (TA Instruments SDT Q600, USA). Here, small microcapsules (5–7 mg) were heated from 23 °C to 800 °C at a rate of 10 °C/min in a nitrogen environment.

#### 2.4.11. UV-Accelerated Aging Test

The RTPUU microcapsule ink obtained using the method described in Section 2.4.7 was coated on white matte art paper using an automatic film applicator (AFA-IV, Shanghai Modern Environment Engineering Technique Co., Ltd., Shanghai, China). After drying in an air oven at 80 °C for 15 min, the RTPUU microcapsule ink coating samples were obtained. These samples were subjected to a UV-accelerated aging test using a 3-A standard solar simulator light source (optical fiber xenon light source CME-303F, light source: 5000 Lu/D65/1000 ± 50 W/m^2^, UV: 3000 ± 100 μW/cm^2^, Beijing Micro Energy Technology Co., Ltd., Beijing, China). The color of the samples was measured and recorded using a luminance colorimeter (CS-150, test mode: interval/300 s, angle: 5°, Konica Minolta Investment Ltd., Shanghai, China) by conducting the following two tests: (1) Color fastness: the real-time tristimulus values recorded in the luminance colorimeter were converted into the L*, a*, and b* color coordinates of the sample; the initial L*, a*, and b* color coordinates of the sample were considered as the base point color coordinates. The sample with the L*, a*, and b* color coordinates was tested under the solar simulator light source for 1/5/10/15/20/25–50 h, and its color coordinates were compared with those of the base point. The color difference Δ*E* was calculated, and the Δ*E* values of different samples were compared under the same testing conditions. (2) Resistance time: in a dark environment, the optical fiber xenon light source was used to simulate a standard solar irradiance of 1000 ± 50 W/m^2^ (refer to the international standard ISO 9845-1:2022 “Solar energy—Reference solar spectral irradiance at the ground at different receiving conditions—Part 1: Direct normal and hemispherical solar irradiance for air mass 1,5” [23]). The measured and recorded ultraviolet irradiance was 3000 ± 100 μW/cm^2^. The angle between the measurement direction of the luminance colorimeter and the irradiation direction of the light source was set to 5°. The sample was placed at the intersection point of the measurement direction and the irradiation direction, and the light source was kept at vertical luminance. The real-time tristimulus values were recorded and converted into the L*, a*, and b* color coordinates of the sample. Δ*E* between the real-time L*, a*, and b* color coordinates and those of the base point was calculated. When Δ*E* remained unchanged, the measurement ended, and the irradiation time required for Δ*E* to become stable was selected as the UV endurance time. All samples were tested at 25 °C and 40% humidity.

#### 2.4.12. Test on Solvent and Acid–Base Resistances

Solvent resistance: Four groups of 0.1 g microcapsules and four groups of 0.1 g RT compositions were weighed and placed in eight glass sample bottles. Then, 10 g each of cyclohexanone, toluene, xylene, and chloroform were added to each bottle. Further, each bottle was sealed with a screw cap and placed on the test bench. After 48 h, the color change and dissolution of the microcapsules and RT compositions were observed.

Acid–base resistance: Three groups of 0.1 g microcapsules and three groups of 0.1 g RT compositions were weighed and placed in six glass sample bottles. Then, 10 g each of 1 M hydrochloric acid, sulfuric acid, and sodium hydroxide were added to each bottle. Further, each bottle was sealed with a screw cap and placed on the test bench. After 48 h, the color change and dissolution of the microcapsules and RT compositions were observed.

## 3. Results and Discussion

### 3.1. Orthogonal Experimental Analysis and Single-Factor Experiment Results

The results of the L_9_ (3^4^) orthogonal experiment on RTPUU microcapsules are presented in Table 3. Sample 6 exhibited the highest encapsulation efficiency (86.54% ± 1.32%). When the N3300-to-L75 shell material mass ratio was 1:4, the core-to-shell material mass ratio was 11:5, the emulsifier concentration was 3.5%, the shear rate during emulsification was 3500 rpm, and the encapsulation efficiency reached the maximum value. The ANOVA results (Table 4) revealed that the shear rate during emulsification, had a significant effect on the encapsulation efficiency, followed by the N3300-to-L75 shell material mass ratio, core-to-shell material mass ratio, and emulsifier concentration. Furthermore, the results showed that the F-ratios of the three factors (the N3300-to-L75 shell material mass ratio, core-to-shell material mass ratio, and emulsifier concentration) were considerably lower than the critical F-value. These three factors demonstrated negligible effect on the test results; thus, an N3300-to-L75 shell material mass ratio of 1:4, a core-to-shell material mass ratio of 11:5, and an emulsifier concentration of 3.5% were considered the optimal choice. Consequently, a single-factor optimization experiment was designed and conducted by varying the shear rate during emulsification.

Based on the results of the orthogonal experiment presented above, the N3300-to-L75 shell material mass ratio was set to 1:4, the core-to-shell material mass ratio was set to 11:5, and the emulsifier concentration was set to 3.5%. The shear rate during emulsification was varied (2000, 2500, 3000, and 4000 rpm). Table 5 presents the encapsulation efficiency determined in the single-factor microcapsule experiments. As the shear rate during emulsification increases, the encapsulation efficiency initially increases and then decreases. The encapsulation efficiency is the highest (93.59% ± 1.53%) at a shear rate of 2500 rpm and the lowest (85.03% *±* 1.14%) at a shear rate of 4000 rpm. Low shear rates produce large oil particles, resulting in an unstable emulsion and low encapsulation efficiency. Alternatively, high shear rates produce small oil particles, causing a thin shell and a small microcapsule diameter [24]. Hence, microcapsules are easily broken, resulting in a low encapsulation rate.

### 3.2. Impact of Shear Rate during Emulsification

#### 3.2.1. PSD of the RTPUU Microcapsules

**Figure 3:**
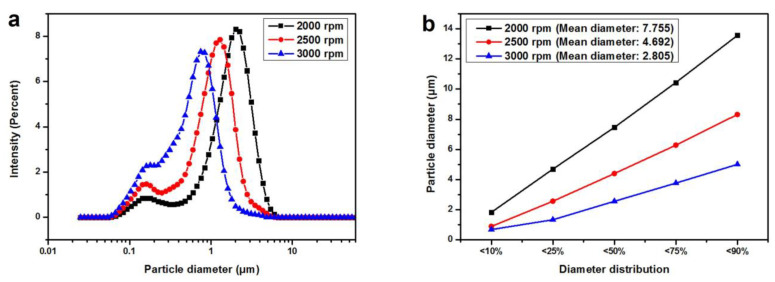
(**a**) PSD curves of the RTPUU microcapsules obtained at shear rates of 2000, 2500, and 3000 rpm; (**b**) Diameter distribution of the RTPUU microcapsules produced under shear rates of 2000, 2500, and 3000 rpm.

The size distribution of the microcapsules was measured by varying the shear rate while keeping all the other factors constant (N3300-to-L75 shell material mass ratio = 1:4, core-to-shell material mass ratio = 11:5, and emulsifier concentration = 3.5%). The results are shown in Figure 3a. As the shear rate increases from 2000 to 3000 rpm, the average particle diameter of microcapsules decreases noticeably, exhibiting a narrow size distribution. Figure 3b shows the diameter distribution of the prepared microcapsules under different shear rates and is presented as a volume distribution. As the shear rate during emulsification increases, the mean diameter of microcapsules gradually decreases from 7.755 to 2.805 μm. This indicates that the mean diameter of the microcapsules is inversely proportional to the shear rate. This is because shear rate controls the equilibrium between the shear and interfacial tensile forces of discrete oil droplets. At a low shear rate, the interfacial tensile force dominates; hence, the droplets become comparatively large. Alternatively, strong shear force at a high shear rate creates small droplets.

#### 3.2.2. Surface Morphology of the RTPUU Microcapsules

**Figure 4:**
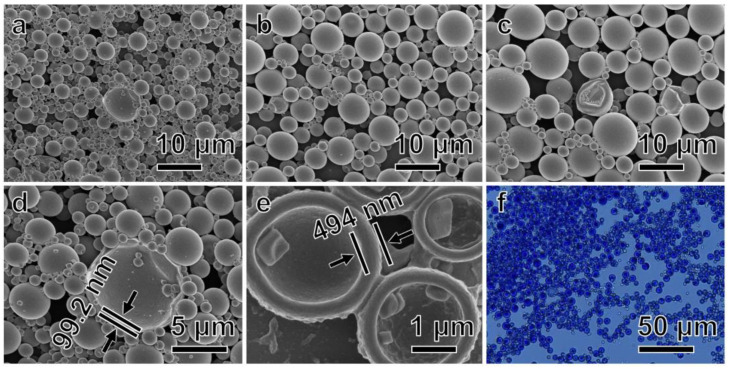
SEM images of the RTPUU microcapsules produced under shear rates of (**a**) 3000; (**b**) 2500; and (**c**) 2000 rpm. (**d**) Cross-sectional SEM image of a ruptured microcapsule in (**a**). (**e**) Cross-sectional SEM image of the RTPUU microcapsules produced under a shear rate of 2500 rpm after fracturing under liquid nitrogen. (**f**) Optical microscopic photograph of the RTPUU microcapsules produced under a shear rate of 2000 rpm.

The SEM images of the microcapsules shown in Figure 4 were used to observe the surface morphological changes in the microcapsule particles at different shear rates during emulsification. Figure 4a shows that some microcapsules are broken, and the shell thickness of a ruptured microcapsule is ~99.2 nm (Figure 4d), indicating that the microcapsules obtained at a shear rate of 3000 rpm have thin shells and poor mechanical properties. However, at lower shear rates, the size of microcapsules increases. Additionally, when the shear rate was decreased to 2000 rpm, some microcapsules (Figure 4c) exhibited an irregular and more concave shape compared with other samples. Furthermore, the optical microscopic photograph (Figure 4f) indicates that microcapsules exhibit a blue color at room temperature (25 °C), and there is no adhesion between the particles. This is probably because, at low shear rates, the microcapsules become unstable and mechanically deformed. The SEM images of the microcapsules at a shear rate of 2500 rpm (Figure 4b) exhibit a regular spherical shape with a dense shell thickness of ~494 nm (Figure 4e), indicating that the RTPUU microcapsules are sufficiently strong to overcome the dispersion process in the ink.

#### 3.2.3. Dispersibility of the RTPUU Microcapsule Powder

Table 6 shows that as the shear rate increases during emulsification, the fineness value of the RTPUU microcapsule powder dispersed in the ink initially decreases and then increases. This is because, at a shear rate of 2000 rpm, the shearing force of the mechanical dispersion disk applied to the oil–water mixture is insufficient. Consequently, the particle size of the oil droplets dispersed in the water phase is very large, resulting in a relatively large particle size of the obtained microcapsules. The fineness value of the microcapsule powder in the ink is 22.5 μm. At a shear rate of 3000 rpm, although the average particle size of microcapsules in suspension decreases, the filtration time increases and the particle passing rate through a 400-mesh sieve decreases; the fineness value of the microcapsule powder in the ink was 15 μm. The fineness value increases to ~2.5 μm compared to a 2500-rpm shear rate. These results indicate that the RTPUU microcapsule powder prepared by increasing the shear rate to 3000 rpm exhibits poor dispersibility in the screen-printing ink, causing agglomeration. This is because when the particle size of microcapsules decreases, the surface energy and aggregation rate increase. After being dispersed in the ink, the microcapsule particles spontaneously aggregate to reduce the specific surface area and become coarse. Generally, to ensure the high-quality of screen-printing products, the fineness value should be less than or equal to 25 μm. The particle sizes of the RTPUU microcapsules obtained using three different shear rates meet the requirements of screen-printing products.

#### 3.2.4. Reversible Thermochromic Properties of the RTPUU Microcapsules

**Figure 5:**
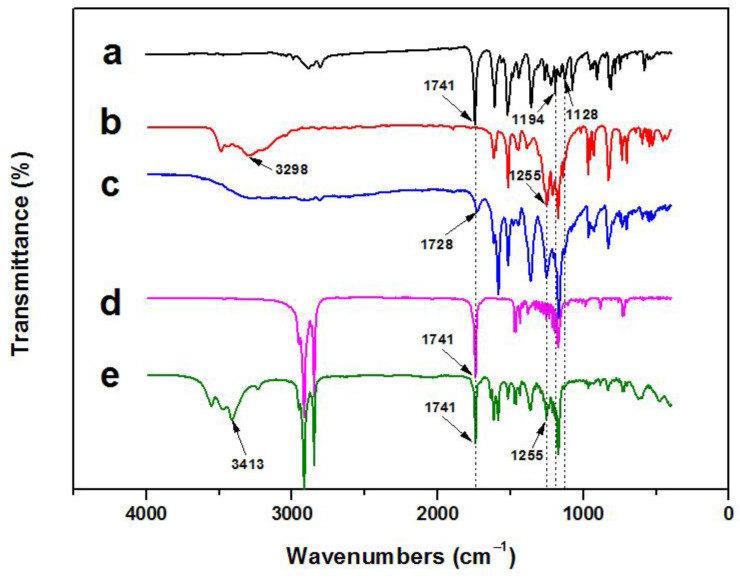
FT-IR spectra of (a) CVL, (b) BPAF, (c) CVL and BPAF compounds, (d) Me-st, and (e) RT compositions of CVL, BPAF, and Me-st.

The FT-IR spectrum of CVL (Figure 5a) shows that the single peak at 1741 cm^−1^ corresponds to the C=O stretching vibration peak of the lactone carbonyl group. Antisymmetric and symmetric stretching vibration absorption peaks of C–O–C in the lactone group are observed at 1194 cm^−1^ and 1128 cm^−1^, respectively. The above three characteristic absorption peaks confirm the presence of a closed lactone ring in CVL.

The above three characteristic peaks of CVL disappeared in the FT-IR spectrum of the CVL and BPAF compounds (Figure 5c), indicating that the lactone ring structure of the compounds no longer existed. At 1728 cm^−1^, a weak absorption peak corresponding to the C=O stretching vibration absorption peak after the lactone ring opens, indicating that CVL and BPAF interact and open the loop to form a conjugated structure, generating a blue chromogen.

The FT-IR spectrum of RT compositions (formed by adding Me-st as a solvent to the CVL and BPAF compounds) (Figure 5e) shows that the broad peak at 3413 cm^−1^ corresponds to the stretching vibration absorption peak of the intermolecular hydrogen O–H bond, and the C–O stretching vibration absorption peak of BPAF (Figure 5b) appears at 1255 cm^−1^. The two characteristic antisymmetric and symmetric stretching vibration absorption peaks of C–O–C of the lactone group in CVL at 1194 cm^−1^ and 1128 cm^−1^ are very weak (almost disappear). The C=O stretching vibration absorption peak at 1741 cm^−1^ corresponds to the ester carbonyl group C=O stretching vibration absorption peak of Me-st (Figure 5d). These results indicate that, when the solvent Me-st is in its solid state at room temperature, the lactone ring in the CVL molecule opens under the effect of BPAF, generating a carboxyl group. By analyzing the FT-IR spectra, thermochromic mechanism of the RT compositions is verified.

The hysteresis width of the discoloration temperature is an important indicator to examine the thermochromic performance of the RTPUU microcapsules. As the hysteresis width of discoloration temperature decreases, the thermochromic sensitivity of the microcapsules increases. Table 7 shows the discoloration temperatures of the RTPUU microcapsule suspension coatings under different shear rates. As the shear rate increases, the hysteresis width of the discoloration temperature initially decreases and then increases. The narrowest hysteresis width of 6.45 °C is obtained under a shear rate of 2500 rpm, indicating good thermochromic performance.

Table 8 shows the color density and color residue of the screen-printing ink containing RTPUU microcapsules prepared at shear rates of 2000, 2500, and 3000 rpm. The color density decreases gradually as the shear rate during emulsification increases. As the shear rate increases, the color residue initially decreases and then increases. The highest color density and color residue (85.44 and 4.59, respectively) are obtained at a shear rate of 2000 rpm. At a shear rate of 3000 rpm, the lowest color density and high color residue are obtained. However, the acceptable color density and color residue values are obtained at a shear rate of 2500 rpm.

**Figure 6:**
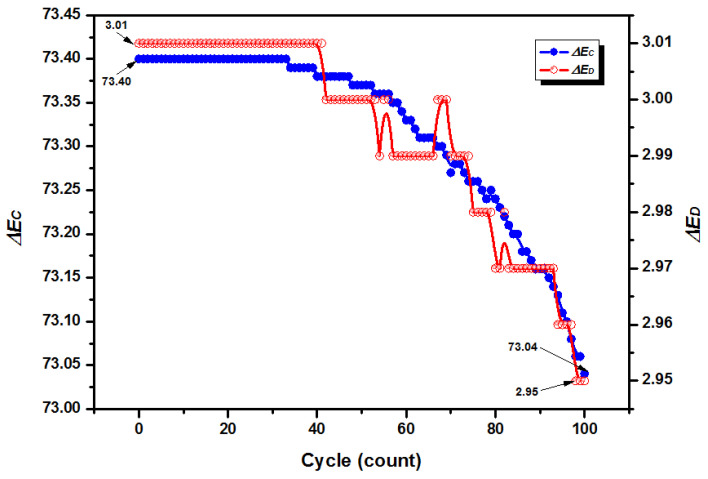
Color density Δ*E_C_* and color residue Δ*E_D_* of a screen-printing ink coating containing RTPUU microcapsules prepared at a shear rate of 2500 rpm under 100 times of repeating temperature change cycles.

To test the reversible thermochromic performance of the RTPUU microcapsules, the screen-printing ink coating containing RTPUU microcapsules were prepared at a shear rate of 2500 rpm and subjected to repeated temperature change cycles. After 100 cycles, the color density Δ*E_C_* (73.04) and color residue Δ*E_D_* (2.95) of the RTPUU microcapsule ink coating maintained over 99.5% and 98.0 % of the initial values (73.40 and 3.01), respectively (Figure 6). Thus, the results exhibited negligible decoloration and retained almost the initial performance, demonstrating their excellent reversibility and cyclic stability at a certain thermochromic transition temperature.

In this study, a shear rate of 2500 rpm, N3300-to-L75 shell material mass ratio of 1:4, core-to-shell material mass ratio of 11:5, and emulsifier concentration of 3.5% were identified as the optimal parameter values for preparing the RTPUU microcapsules. Based on the above parameter values, an RTPUU microcapsule sample with high encapsulation efficiency, dense shell, satisfactory microsize, good dispersibility, and good reversible thermochromic properties (such as high color density, low color residue value, narrow hysteresis width of the discoloration temperature, excellent reversibility, and cyclic stability) was prepared. Owing to these characteristics, the RTPUU microcapsules are suitable for use in screen-printing ink and facilitate its processing as well as dispersion in ink for subsequent applications.

### 3.3. Polymerization Process for the Formation of the RTPUU Microcapsules

#### 3.3.1. Interfacial Polymerization Encapsulation Process

**Figure 7:**
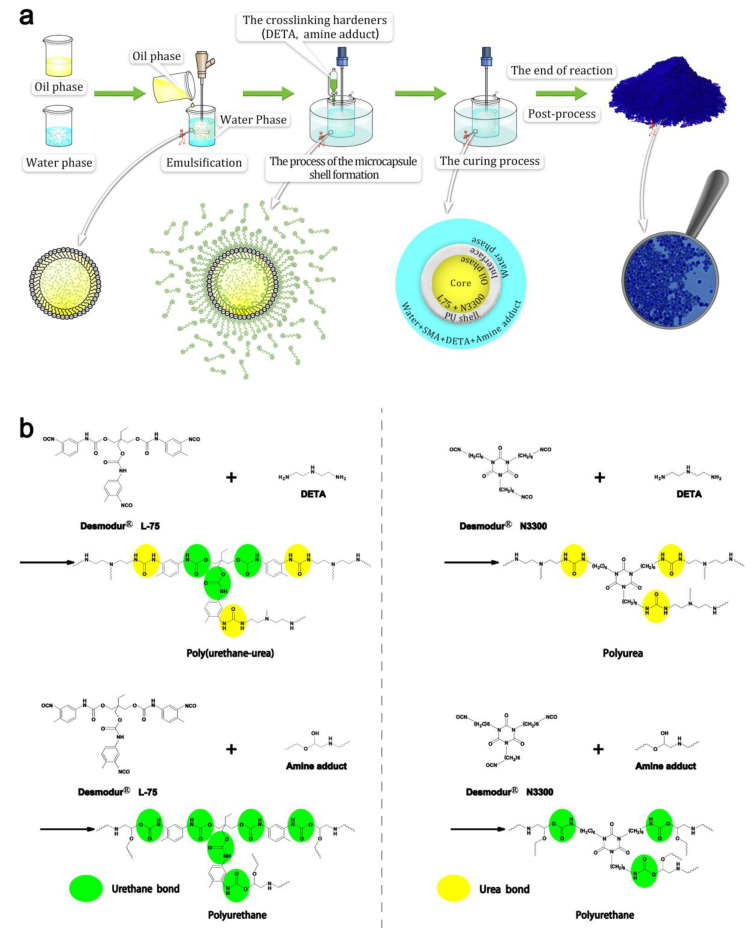
Schematics of the polymerization process used to produce the RTPUU microcapsules: (**a**) The encapsulation process based on interfacial polymerization; (**b**) The reaction scheme used to form the microcapsule shell [18].

Interfacial polymerization encapsulation usually includes three steps: emulsification of the core materials (oil phase), microcapsule shell formation, and curing. Figure 7 shows the polymerization process employed to produce the RTPUU microcapsules. A homogenous oil phase containing RT composition, shell material, and cosolvent was prepared and added to the water phase containing an SMA surfactant. Mechanical emulsification was used to produce a stable oil-in-water (O/W) emulsion. During this process, the surfactant molecules trimly covered the surfaces of the oil-phase droplets. The SMA macromolecules were negatively charged by adjusting the pH value of the emulsion system to below 4.5. These macromolecules were subsequently attached to the interface, where their lipophilic ends extend into the interior of the oil droplets, and the hydrophilic ends of the carboxyl groups extend into the water phase. Hence, the surfaces of the oil droplets were negatively charged. Subsequently, crosslinking hardeners (DETA and amine adduct) were added to the uniform and stable emulsion system. The crosslinking hardeners contained –NH_2_, –NH–, and –NH–CH_2_–CH(OH)–O– groups in their structure, which dissolved in the aqueous acidic emulsion and became positively charged. These positively charged crosslinking hardeners were then adsorbed onto the surface of the oil droplets because of the attraction between positive and negative charges. Meantime, some shell material molecules (Desmodur^®^ L-75 prepolyurethane and N3300 HDI trimer) were diffused onto the surface of the oil droplets via a diffusion effect. Under certain temperatures and catalyst conditions, the shell material molecules within the oil droplets continuously diffused to the O/W interface until the polymerization reaction with the crosslinking hardeners was completed. Further curing was performed to form solid microcapsules with well-defined shell strength.

#### 3.3.2. FT-IR Analysis of the RTPUU Microcapsules

**Figure 8:**
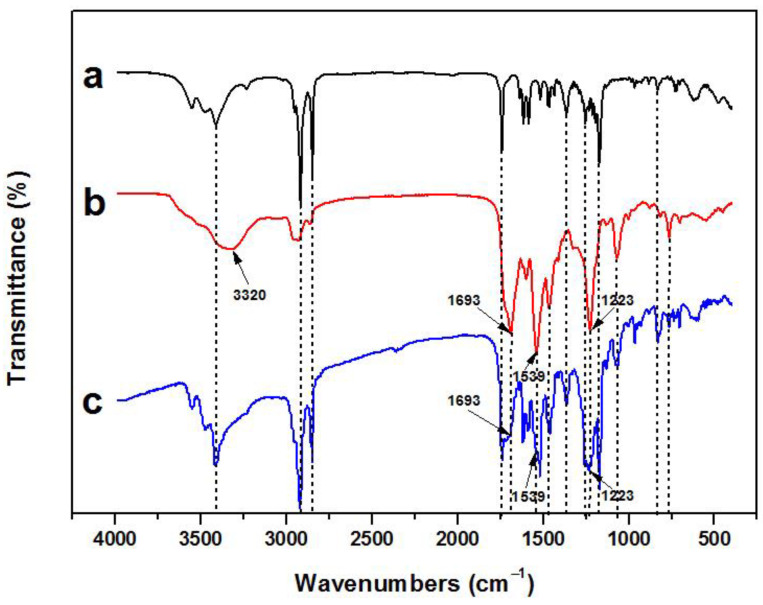
FT-IR spectra of (a) the RT compositions of CVL, BPAF, and Me-st, (b) PUU microcapsule shell, and (c) RTPUU microcapsules.

The FT-IR spectrum of the microcapsule shell (Figure 8b) shows that the characteristic absorption peak of the isocyanate group (–N=C=O) is not observed (at 2272 cm^−1^), indicating that the –N=C=O in Desmodur^®^ L75 and N3300 were fully reacted. The absorption peak at 3320 cm^−1^ and 1693 cm^−1^ correspond to the N–H bond stretching vibration and C=O stretching vibration peak, respectively. Furthermore, the absorption peak at 1539 cm^−1^ corresponds to the N–H bending vibration of amide II [25], which is generated by the reaction between –OH (or –NH_2_) and –N=C=O. The abovementioned three peaks are the characteristic vibration peaks of the urethane bond (–NH–CO–O–) and urea bond (–NH–CO–NH–) in poly(urethane-urea). Additionally, the absorption peak at 1223 cm^−1^ corresponds to the C–O–C stretching vibration of the urethane bonds in poly(urethane-urea). These results indicate that the poly(urethane-urea) shells are generated.

Figure 8a shows the FT-IR spectrum of the RT compositions shown in Figure 5e, which has been analyzed above. A comparison of the three FT-IR spectra shown in Figure 8 indicates that the RT compositions are encapsulated, and the FT-IR spectrum of microcapsules (Figure 8c) exhibits the characteristic peaks of the RT compositions and shell, proving that the microcapsule with the poly(urethane-urea) shell can effectively encapsulate the RT compositions.

### 3.4. Phase-Change Characteristics of the RTPUU Microcapsules

**Figure 9:**
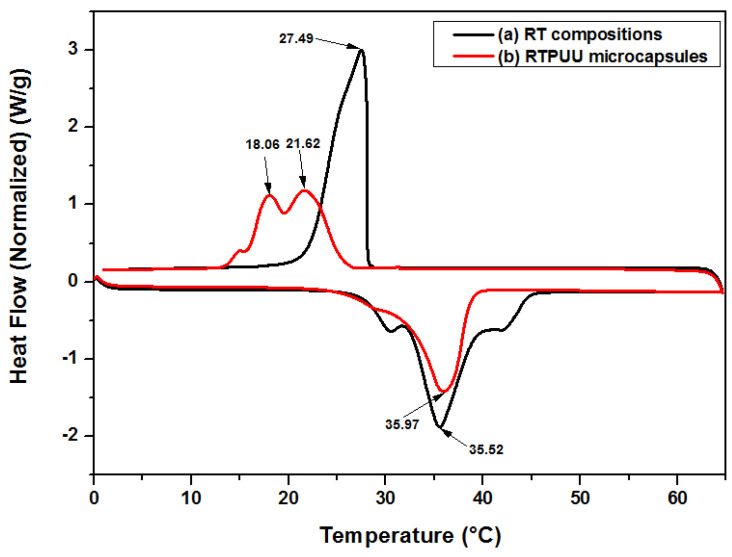
DSC curves of (a) the RT compositions of CVL, BPAF, and Me-st and (b) RTPUU microcapsules without B225 antioxidant.

The melting and crystallization curves of the RT compositions and RTPUU microcapsules without B225 antioxidant are shown in Figure 9, and the corresponding DSC data are presented in Table 9. The DSC curves in Figure 9 exhibit obvious melting and crystallization peaks, indicating that the RT compositions and RTPUU microcapsules have certain phase-change energy storage capacities. The melting peak temperatures of the RT compositions and microcapsules are close, and the melting and crystallization enthalpies of the microcapsules exhibit a drop at these temperatures.

Based on the data analysis presented in Table 9 and Table 10, we found that in the discoloration process of microcapsules without B225, the melting peak temperature *T_peak,H_* (see Table 9) is directly correlated with the complete decoloration temperature *T*_4_ (see Table 10). Furthermore, the cooling curve of the microcapsules exhibits two obviously separated crystallization peaks at 21.62 °C and 18.06 °C (α crystallization and β crystallization peaks, respectively), corresponding to the heterogeneous and homogeneous nucleation processes of the microencapsulated RT compositions, respectively [26,27,28]. The two separated crystallization peaks are below the crystallization peak of RT compositions at 27.49 °C, implying that crystallization becomes difficult. This is mainly caused by the confinement of the microcapsule size [29] and thermal resistance of the shell [30]. This supercooling phenomenon is considered to be closely related to the hysteresis of the discoloration temperature of the microcapsules. For example, during the discoloration process of the microcapsules without B225, the complete coloration temperature *T*_1_ (see Table 10) corresponds to the temperature at which the β crystallization peak *T_peak,c_* _(*β*)_ (see Table 9) appears as these two temperatures are very close in value.

Moreover, the estimated core content *C_e_* and encapsulation efficiency *E_e_* are introduced to evaluate the encapsulation ratio of the RT compositions. *C_e_* and *E_e_* can be calculated from the DSC results using the following Equations (4) and (5).

(4) $$C_{e}\;(\%)=\frac{{\;(\Delta Hm)}_{micro.}}{{\Delta H}_{0}}\times 100$$

(5) $$E_{e}\;(\%)=\frac{{\;(\Delta H_{m})}_{micro.}}{\;\Delta H_{0}\times \frac{W_{0}}{W_{s}}}\times 100$$

where (Δ*Hm*)*_micro_*. is the melting enthalpy of the microcapsules obtained from DSC measurements, Δ*H*_0_ is the melting enthalpy of the initially added RT compositions (i.e., Δ*H*_0_ = 146 J/g from DSC measurements), *W*_0_ is the weight of fed RT compositions, and *W_s_* is the weight of RT compositions and shell materials in the feed [31,32].

The values of *C_e_* and *E_e_* calculated using Equations (4) and (5) are 62.38% and 90.74%, respectively, which are close to the actual values of *C_a_* (65.11% ± 0.26%) and *E_a_* (93.59% ± 1.53%).

### 3.5. Thermal Stability of the RTPUU Microcapsules

**Figure 10:**
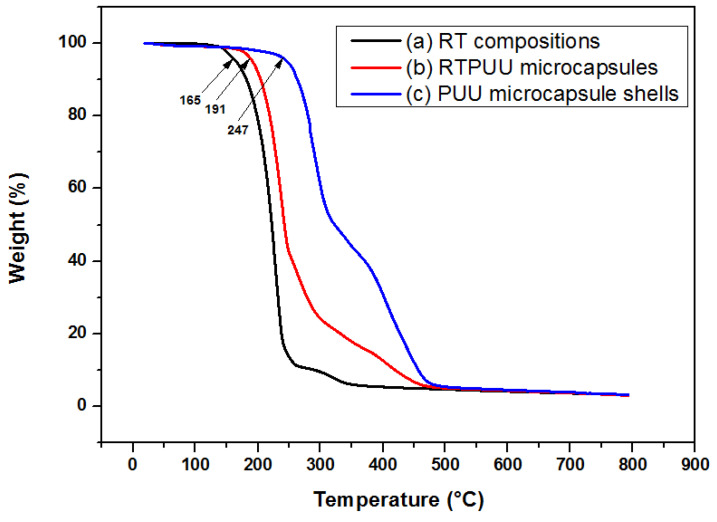
TGA curves of (a) the RT compositions, (b) RTPUU microcapsules, and (c) PUU microcapsule shell.

The TGA curves of the RT compositions, RTPUU microcapsules, and PUU microcapsule shell are shown in Figure 10. Generally, for these three samples, a 5 wt% mass loss temperature is considered as a heat-resistant temperature during the heating process [33]. Figure 10a shows that the mass loss process of the RT compositions starts at ~165 °C and ends at ~368 °C. Figure 10c shows that the mass loss process of the PUU shells starts at ~247 °C and ends at ~494 °C. Therefore, the onset temperature for weight loss of the PUU shell is much higher than that of the RT compositions, demonstrating that the thermal stability of the PUU shell is higher than that of the RT compositions. Compared with the RT compositions, the RTPUU microcapsules (Figure 10b) start to lose weight at a high temperature (~191 °C), and this process continues up to ~494 °C. The above results demonstrate that the PUU shell acts as a physical protective barrier and prevents the escape of volatile products during the heating process, and the thermal stability of microcapsules is improved.

### 3.6. UV-Aging Resistance of the RTPUU Microcapsules

An antioxidant terminates chain reactions in a polymer by trapping free radicals or decreasing the chain reaction rate by decomposing peroxides, thereby improving the antiaging properties of the polymer [34]. The B225 antioxidant is a unique blend of a main antioxidant 110 (phenolic antioxidant) and a secondary antioxidant 168 (phosphate co-stabilizer) at a 1:1 ratio, and it was added to the RTPUU microcapsules to investigate their impact on the UV-aging resistance and thermochromic properties.

**Figure 11:**
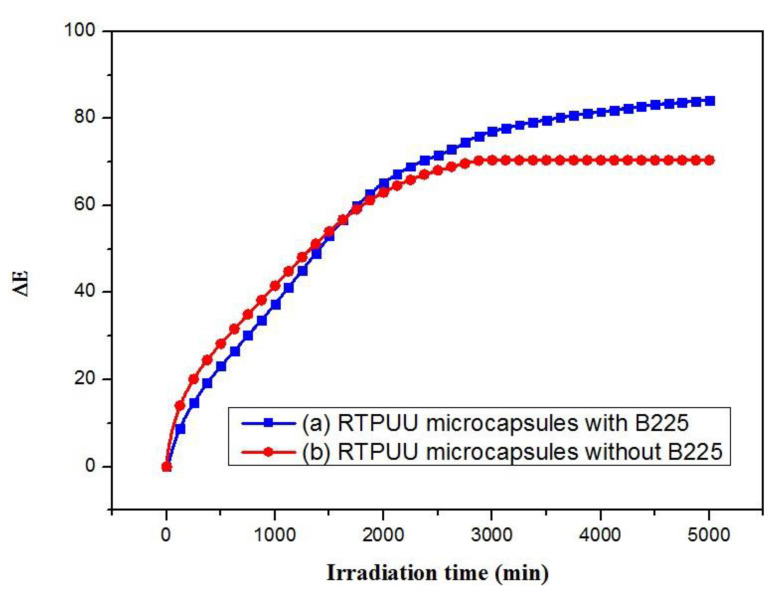
Curves of Δ*E* vs. irradiation time of the RTPUU microcapsules (a) with and (b) without B225 antioxidant.

Figure 11 shows that Δ*E* of the RTPUU microcapsules without B225 is stable and unchanged after UV irradiation for 2895 min under 3000 ± 100-μW/cm^2^ aging condition. For comparison, the Δ*E* of the RTPUU microcapsules with B225 becomes stable after irradiation for 5015 min. Thus, B225 antioxidant greatly improves the UV-aging resistance of the RTPUU microcapsules. Although excellent UV-aging resistance was obtained in this study, an improvement in the weatherability is desired. This can be achieved through further research.

Table 10 and Table 11 show that the RTPUU microcapsules with and without B225 antioxidant are blue at room temperature. When heated to 31 °C or higher, the color disappeared and the microcapsules became colorless. Compared to the RTPUU microcapsules without B225, those with B225 exhibited the same level of color density and lower color residue value, indicating good coloration and decoloration performance. The hysteresis width of discoloration temperature of the microcapsules with B225 is reduced by 1.9 °C, indicating improved discoloration hysteresis and good thermochromic performance.

These results indicate that microcapsules with B225 antioxidant exhibit excellent UV-aging resistance and thermochromic properties, making them suitable for industrial applications. The UV-aging resistance and covering ability were significantly improved, and fading was reduced once the RT compositions were encapsulated, indicating the improved thermochromic effect and stability of the RT compositions.

### 3.7. Solvent and Acid–Base Resistances of the RTPUU Microcapsules

Table 12 shows that the RT compositions have very weak solvent and acid–base resistances. The RT compositions dissolved quickly in solvents shown in Table 12, and the original blue compositions became colorless. Furthermore, the RT compositions faded after their immersion in acid–base solutions shown in Table 12 for 48 h. The solvent and acid–base resistances of RTPUU microcapsules were considerably higher than those of the RT compositions. After 48 h, the microcapsules remained blue and did not dissolve in the solvent or acids/bases. A transparent and dense poly(urethane-urea) shell, which was formed on the surface of the RT compositions, prevented the penetration of solvent and acid–base molecules; therefore, the RT compositions could maintain their color-change performance.

### 3.8. Real-Time Application of the RTPUU Microcapsules

The RTPUU microcapsules of samples (I)–(IV) shown in Table 13 were prepared according to the abovementioned methods, with the exception that the components in each RT composition and their corresponding amounts were changed as shown in the table. The discoloration temperatures were also measured using the aforementioned measurement methods.

The ink was prepared by uniformly dispersing 10 parts of the RTPUU microcapsule powder into 90 parts of varnish for silk screen printing (SS-777, Toyo East Ocean ink). Using this ink, several anticounterfeiting patterns were screen-printed on white paper using a 100-mesh screen plate to create an RT anticounterfeiting mark.

**Figure 12:**
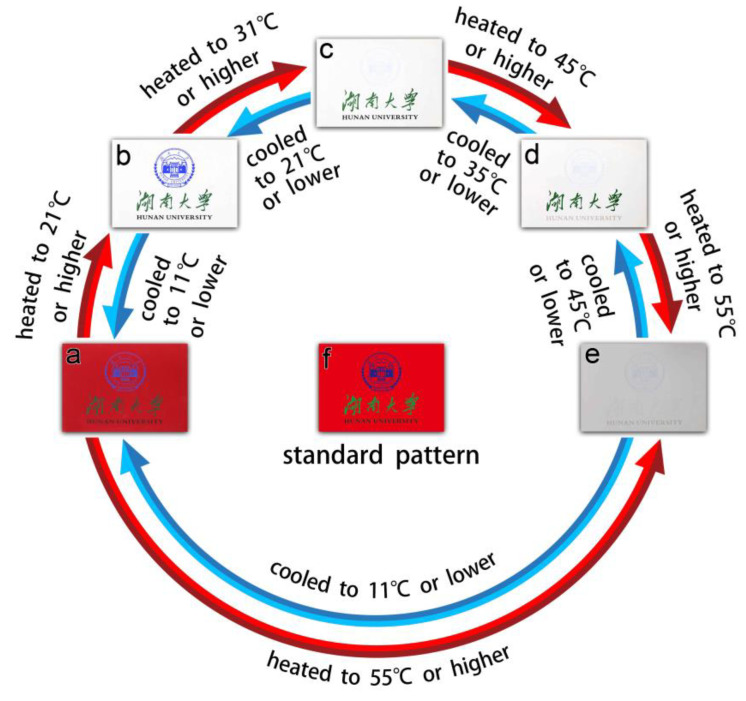
Diagrams showing the reversible thermochromic performance of an anticounterfeiting pattern obtained from four different colored inks containing RTPUU microcapsules.

Herein, the anticounterfeiting pattern (Figure 12f) consisted of a red background (21 °C), a blue logo (31 °C), black English words (45 °C), and green Chinese characters (55 °C). At room temperature (25 °C), the blue logo, black English words, and green Chinese characters could be observed visually (see Figure 12b). However, the blue logo turned colorless when the mark was heated to 31 °C or higher (see Figure 12c). Similarly, heating the mark at 45 °C or higher caused the black English words to be colorless (see Figure 12d). Furthermore, the green Chinese characters became colorless when heated to 55 °C or higher (see Figure 12e). However, upon cooling, the green Chinese characters, black English words, and blue logo regained their visibility at ≤45 °C, ≤35 °C, and ≤21 °C, respectively. Additionally, the red background appeared colorless at room temperature (25 °C). However, upon cooling to 11 °C or lower, the red background became visually recognizable again, but it turned colorless once more when the mark was heated to 21 °C or higher (see Figure 12a,b). This behavior was reproducible.

Thus, the RTPUU microcapsules exhibited a unique reversible thermal memory function and strong color contrast between the cooling and heating conditions. These properties will make them ideal for achieving distinctive anticounterfeiting characteristics when incorporated into inks.

## 4. Conclusions

In this study, RTPUU microcapsules for the encapsulation of RT compositions were successfully prepared via interfacial polymerization using a poly(urethane-urea) (PUU) shell. These microcapsules can be used in anticounterfeiting technical products. The orthogonal experiment showed that an N3300-to-L75 shell material mass ratio of 1:4, core-to-shell material mass ratio of 11:5, and emulsifier concentration of 3.5% were the optimal parameter values for preparing RTPUU microcapsules. The shear rate during emulsification was found to be the most influential factor affecting the encapsulation efficiency of the microcapsules. A single-factor optimization experiment was conducted with shear rate as the variable during emulsification. At a shear rate of 2500 rpm, the microcapsule performance was found to be the best. Then, the core content and encapsulation efficiency of the RTPUU microcapsules reached their highest values, their particle diameter exhibited a narrow size distribution, and the particles exhibited a regular spherical shape with a dense shell. Furthermore, the dispersibility of the RTPUU microcapsule powder in a screen-printing ink was good, the hysteresis width of the discoloration temperature of the RTPUU microcapsules exhibited their narrowest value, and their color density and color residue values were acceptable. Moreover, the RTPUU microcapsules exhibited good thermal stability, UV-aging, and solvent and acid–base resistances.

The RTPUU microcapsules were added to the varnish of a silk screen-printing ink to obtain a reversible thermochromic anticounterfeiting mark, which exhibited a unique reversible thermal memory function and strong color contrast between the cooling and heating conditions. This result reveals that the microcapsules could be used to achieve distinctive anticounterfeiting ink characteristics. The results also laid the foundation for using thermochromic coatings in anticounterfeiting technical products.

## Figures and Tables

**Table 1:** Influential factors and levels of the orthogonal experiment.

Level	N3300-to-L75 Shell Material Mass Ratio	Core-to-Shell Material Mass Ratio	Emulsifier Concentration (%)	Shear Rate (rpm)
1	1:3.5	11:4	3.5	1500
2	1:4	11:4.5	4	3500
3	1:5	11:5	5	4500

**Table 2:** Orthogonal experimental design with four factors and three levels.

Level	N3300-to-L75 Shell Material Mass Ratio	Core-to-Shell Material Mass Ratio	Emulsifier Concentration (%)	Shear Rate (rpm)
1	1:3.5	11:4	3.5	1500
2	1:3.5	11:4.5	4	3500
3	1:3.5	11:5	5	4500
4	1:4	11:4	4	4500
5	1:4	11:4.5	5	1500
6	1:4	11:5	3.5	3500
7	1:5	11:4	5	3500
8	1:5	11:4.5	3.5	4500
9	1:5	11:5	4	1500

**Table 3:** Orthogonal experimental results.

Exp. No.	N3300-to-L75 Shell Material Mass Ratio	Core-to-Shell Material Mass Ratio	Emulsifier Concentration (%)	Shear Rate (rpm)	E_a_ ^1^ (%)
1	1:3.5	11:4	3.5	1500	75.36 ± 1.04
2	1:3.5	11:4.5	4	3500	84.46 ± 0.97
3	1:3.5	11:5	5	4500	75.18 ± 0.99
4	1:4	11:4	4	4500	74.70 ± 0.61
5	1:4	11:4.5	5	1500	76.86 ± 1.03
6	1:4	11:5	3.5	3500	86.54 ± 1.32
7	1:5	11:4	5	3500	83.42 ± 1.14
8	1:5	11:4.5	3.5	4500	74.87 ± 0.81
9	1:5	11:5	4	1500	74.96 ± 0.93
Mean _(1)_ ^2^	78.333	77.827	78.923	75.727	-
Mean _(2)_	79.367	78.730	78.040	84.807	-
Mean _(3)_	77.750	78.893	78.487	74.917	-
Range ^3^	1.617	1.066	0.883	9.890	-

^1^ The actual core content *C_a_* and the encapsulation efficiency *E_a_* were obtained using Equations (1) and (2), respectively. All experiments were conducted thrice, and the results were expressed as mean ± SD (standard deviation) values. The Orthogonality Experiment Assistant II software V3.1 was used to evaluate the statistical experimental design. ^2^ Mean _(i)_ is the average of *E_a_* at the *i*-th level, (*i* = 1, 2, 3…). ^3^ Range is the difference between the maximum and minimum values of the Mean _(i)_ of certain factors, (*i* = 1, 2, 3…).

**Table 4:** Analysis of variance (ANOVA) results [6,19].

Factor	Sum of Squares	Degree of Freedom	F-Ratio	Critical F-Value	Significance (α = 0.10)
N3300-to-L75 shell material mass ratio	4.022	2	0.086	3.113	-
Core-to-shell material mass ratio	1.980	2	0.042	3.113	-
Emulsifier concentration (%)	1.170	2	0.025	3.113	-
Shear rate (rpm)	180.915	2	3.847	3.113	*
Error value	188.090	8	-	-	-

* means that the effect is significant in statistics.

**Table 5:** List of materials, *C_a_*, and *E_a_* in single-factor experiments.

Exp. No.	Shear Rate (rpm)	Shell Materials		Core Materials	Emulsifier Concentration (%)	C_a_ (%)	E_a_ (%)
		N3300 (g)	L75 (g)	CVL: 8 g + BPAF: 20 g + Me-st: 80 g + B225: 2 g			
10	2000	10	40	110	3.5	63.10 ± 0.42	90.78 ± 1.23
11	2500	10	40	110	3.5	65.11 ± 0.26	93.59 ± 1.53
12	3000	10	40	110	3.5	61.65 ± 0.15	88.62 ± 1.31
13	4000	10	40	110	3.5	59.16 ± 0.28	85.03 ± 1.14

**Table 6:** Fineness values of the RTPUU microcapsule powder in a screen-printing ink.

Shear Rate (rpm)	Suction Filtration Time (Min)	Particles Passing Rate through a 400-Mesh Sieve (% of Mass)	Fineness Values of the Ink (μm)
2000	≤20	≤97%	22.5
2500	≤26	≤95%	12.5
3000	≤38	≤90%	15.0

**Table 7:** Discoloration temperature of the RTPUU microcapsule suspension coatings.

Shear Rate (rpm)	Color Change	Discoloration Temperature (°C)						
		T_1_	T_2_	T_3_	T_4_	T_C_	T_D_	ΔT
2000	Blue ⇌ colorless	18.50	28.50	27.50	36.00	23.50	31.75	8.25
2500	Blue ⇌ colorless	18.30	27.80	25.20	33.80	23.05	29.50	6.45
3000	Blue ⇌ colorless	18.00	28.20	26.50	35.50	23.10	31.00	7.90

**Table 8:** Color density Δ*E_C_* and color residue Δ*E_D_* of the RTPUU microcapsule ink coatings.

Sample	State	L*	A*	B*	dL*	Da*	Db*	ΔE_C_	ΔE_D_
Matte art paper	Benchmark sample	92.71	−1.12	0.35	0	0	0	-	-
2000 rpm	Colored (blue)	43.96	24.08	−65.14	−48.75	25.19	−65.49	85.44	-
2500 rpm	Colored (blue)	50.16	15.26	−57.18	−42.55	16.38	−57.53	73.40	-
3000 rpm	Colored (blue)	53.59	10.46	−52.69	−39.13	11.58	−53.04	66.92	-
2000 rpm	Decolored (colorless)	88.96	−3.54	−0.68	−3.76	−2.42	−1.03	-	4.59
2500 rpm	Decolored (colorless)	90.06	−2.38	−0.32	−2.65	−1.26	−0.68	-	3.01
3000 rpm	Decolored (colorless)	88.87	−3.34	−0.01	−3.84	−2.23	−0.36	-	4.45

**Table 9:** DSC data of the RT compositions and RTPUU microcapsules without B225 antioxidant.

Sample	Heating Process (°C)		ΔH_m_ ^1^ (J/g)	Cooling Process (°C)		ΔHc ^2^ (J/g)	C_e_ (%)	E_e_ (%)
	T_onset,H_	T_peak,H_		T_onset,C_	T_peak,C_			
RT compositions	31.78	35.52	146	28.06	27.49	130.92	-	-
RTPUU microcapsules	31.79	35.97	91.078	25.45	21.62 (*α*) ^3^ 18.06 (*β*) ^4^	91.153	62.38	90.74

^1^ ΔH_m_: enthalpy on the DSC heating curve; ^2^ ΔH_c_: enthalpy on the DSC cooling curve; ^3^ T_peak,C (α)_: alpha peak temperature on the DSC cooling curve; and ^4^ T_peak,C (β)_: beta peak temperature on the DSC cooling curve.

**Table 10:** Discoloration temperature of the RTPUU microcapsule suspension coatings with and without B225 antioxidant.

Sample	Color Change	Discoloration Temperature (°C)						
		T1	T2	T3	T4	T_C_	T_D_	ΔT
RTPUU microcapsules with B225	Blue ⇌ colorless	18.30	27.80	25.20	33.80	23.05	29.50	6.45
RTPUU microcapsules without B225	Blue ⇌ colorless	17.50	27.60	26.80	35.00	22.55	30.90	8.35

**Table 11:** Color density Δ*E_C_* and color residue Δ*E_D_* of the RTPUU microcapsules with and without B225 antioxidant.

Sample	State	L*	A*	B*	dL*	Da*	Db*	ΔE_C_	ΔE_D_
Matte art paper	Benchmark sample	93.45	−1.20	0.76	0	0	0	-	-
RTPUU microcapsules with B225	Colored (blue)	32.42	42.09	−73.33	−61.03	43.28	−74.09	105.29	-
RTPUU microcapsules without B225	Colored (blue)	33.77	38.98	−72.77	−59.68	40.18	−73.53	102.87	-
RTPUU microcapsules with B225	Decolored (colorless)	90.35	−2.48	−2.36	−3.10	−1.28	−3.12	-	4.57
RTPUU microcapsules without B225	Decolored (colorless)	88.53	−2.77	−2.51	−4.92	−1.57	−3.27	-	6.12

**Table 12:** Results of tests on the solvent and acid–base resistance of the RT compositions and RTPUU microcapsules.

Reagent	Dissolution		Color Change	
	RT Compositions	RTPUU Microcapsules	RT Compositions	RTPUU Microcapsules
Benzene	Dissolved	Undissolved	Colorless	Invariant
Toluene	Dissolved	Undissolved	Colorless	Invariant
Xylene	Dissolved	Undissolved	Colorless	Invariant
Chloroform	Dissolved	Undissolved	Colorless	Invariant
1 mol/L HCl	Undissolved	Undissolved	Faded	Invariant
1 mol/L H_2_SO_4_	Undissolved	Undissolved	Faded	Invariant
1 mol/L NaOH	Undissolved	Undissolved	Faded	Invariant

**Table 13:** Components and amounts of the RT compositions in four different colored RTPUU microcapsules and their discoloration temperatures.

Sample	RT Compositions (Components and Amounts)			Discoloration Temperature (°C)				
	Leuco Dye	Developer	Solvent	T1	T2	T3	T4	ΔT
(I) 21 °C Red	Red 520 ^1^: 5.50 parts	BPAF: 11.00 parts	Me-st: 10.00 parts	9.00	18.30	11.00	21.20	2.45
			Me-pa ^4^: 40.00 parts					
(II) 31 °C Blue	CVL: 5.00 parts	BPAF: 12.50 parts	Me-st: 50.00 parts	18.30	27.80	25.20	33.80	6.45
(III) 45 °C Black	Black-15 ^2^: 6.25 parts	BPAF: 6.25 parts	Me-st: 6.25 parts	30.70	39.20	41.70	47.20	9.50
			Me-be ^5^: 50.00 parts					
(IV) 55 °C Green	Green 300 ^3^: 2.65 parts	BPAF: 5.25 parts	Me-li ^6^: 56.00 parts	41.00	48.60	47.50	55.80	6.85

^1^ Red 520, CAS: 4228-32-0; ^2^ Black-15, CAS: 36431-22-8; ^3^ Green 300, CAS: 34372-72-0; ^4^ Me-pa: Methyl palmitate; ^5^ Me-be: Methyl behenate; and ^6^ Me-li: Methyl lignocerate.

## Data Availability

Data available on request from the authors.
